# *catena*-Poly[[(3-methyl­pyridine)­copper(I)]-μ-cyanido-copper(I)-μ-cyanido]

**DOI:** 10.1107/S1600536811028509

**Published:** 2011-07-23

**Authors:** Jin-Biao Cai, Ting-Ting Chen, Ze-Ying Xie, Hong Deng

**Affiliations:** a School of Chemistry and Environment, South China Nomal University, Guangzhou 510006, People’s Republic of China

## Abstract

In the title complex, [Cu_2_(CN)_2_(C_6_H_7_N)]_*n*_, there are two copper atoms with different coordination environments. One Cu atom (Cu1) is linked to the two cyanide ligands, one N atom from a pyridine ring while the other (Cu2) is coordinated by the two cyanide ligands in a slightly distorted tetra­hedral geometry and linked to Cu1, forming a triangular coordination environment. The Cu atoms are bridged by bidentate cyanide ligands, forming an infinite Cu–CN chain. One cyanide ligand is equally disordered over two sets of sites, exchanging C and N atoms coordinated to both metal atoms. However, one cyanide group is not disordered and it coordinates to Cu1 *via* the N atom whereas its C-atom counterpart coordinates Cu2. The 3-methyl­pyridine (3MP) ligand coordinates through the N atom to Cu1 as a terminal ligand, which originates from decyanation of 3-pyridyl­acetonitrile under hydro­thermal conditions. Adjacent Cu–CN chains are inter­connected through Cu⋯Cu inter­actions [2.8364 (10) Å], forming a three-dimensional framework.

## Related literature

For applications of coordination polymers, see: Gu & Xue (2007 ▶); Cheng *et al.* (2007 ▶); Ley *et al.* (2010 ▶); Etaiw *et al.* (2009 ▶); Li *et al.* (2009 ▶).
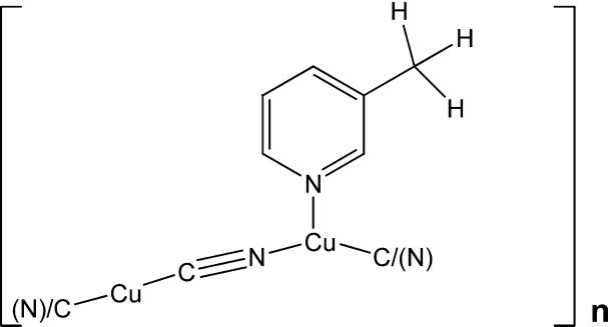


## Experimental

### 

#### Crystal data

[Cu_2_(CN)_2_(C_6_H_7_N)]
*M* *_r_* = 272.27
Monoclinic, 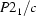

*a* = 9.3027 (18) Å
*b* = 12.090 (2) Å
*c* = 8.8738 (17) Å
β = 105.927 (2)°
*V* = 959.7 (3) Å^3^
*Z* = 4
Mo *K*α radiation
μ = 4.38 mm^−1^
*T* = 296 K
0.15 × 0.12 × 0.10 mm

#### Data collection

Bruker SMART APEX CCD diffractometer
Absorption correction: multi-scan (*SADABS*; Bruker, 2004 ▶) *T*_min_ = 0.559, *T*_max_ = 0.668
4802 measured reflections
1725 independent reflections
1396 reflections with *I* > 2σ(*I*)
*R*_int_ = 0.035

#### Refinement

*R*[*F* ^2^ > 2σ(*F* ^2^)] = 0.042
*wR*(*F* ^2^) = 0.118
*S* = 1.03
1725 reflections
107 parameters
2 restraints
H-atom parameters constrained
Δρ_max_ = 0.76 e Å^−3^
Δρ_min_ = −0.77 e Å^−3^

### 

Data collection: *APEX2* (Bruker, 2004 ▶); cell refinement: *SAINT* (Bruker, 2004 ▶); data reduction: *SAINT*; program(s) used to solve structure: *SHELXS97* (Sheldrick, 2008 ▶); program(s) used to refine structure: *SHELXL97* (Sheldrick, 2008 ▶); molecular graphics: *SHELXTL* (Sheldrick, 2008 ▶); software used to prepare material for publication: *SHELXTL*.

## Supplementary Material

Crystal structure: contains datablock(s) I, global. DOI: 10.1107/S1600536811028509/kp2324sup1.cif

Structure factors: contains datablock(s) I. DOI: 10.1107/S1600536811028509/kp2324Isup2.hkl

Additional supplementary materials:  crystallographic information; 3D view; checkCIF report

## Figures and Tables

**Table 1:** Selected bond lengths (Å)

N1—Cu1	2.057 (2)
C7—Cu2	1.839 (4)
C8—Cu1	1.886 (4)
C9—Cu2	1.838 (5)
Cu1—N2	1.891 (4)

**Fig. 1.**
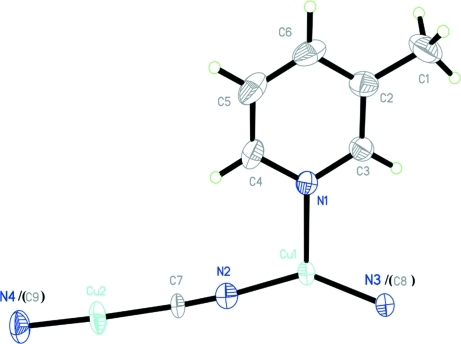
The asymmetric unit of the title complex. Non-H atoms are shown as 50% probability displacement ellipsoids. Two of the cyano ligands N3/(C8) and N4/(C9) are disordered over two sites with occupancy 0.5, each.

## supplementary crystallographic information

### Comment

Much attention has been focused on the rational design and synthesis of coordination polymers due to their intriguing structural features as well as potential applications in catalysis, fluorscence, and as chemical sensors (Gu *et al.*, 2007; Cheng *et al.*, 2007; Ley *et al.*, 2010; Etaiw *et al.*, 2009). Some polymers with rigid ligands such as isonicotinic acid has been reported (Li *et al.*, 2009). A cyano group is a well bridging ligand, which plays an important role in assembling of polymers acting as a monodentate, bidentate or tridentate ligand (Ley *et al.*, 2010). A careful review of the literature suggests that 3-methylpyridine(3MP) use as a ligand to construct metal coordination framework has not been reported yet. Herein, we report the title complex synthesised by the reaction of cuprous cyanide and 3PAT ligands under hydrothermal conditions. Cu1 is coordinated by two cyano ligands, one nitrogen atom from pyridine ring and Cu2 centre with the Cu···Cu distance of 2.836 (4) Å, forming a slightly distorted tetrahedral coordination. Cu2 is coordinated by a carbon atom from one cyano ligand, whereas the second coordination sites is occupied either by N or C atoms (due to the disorder of  ligand), and Cu1 centre forming a triangular coordination environment (Fig. 1, Table 1). The adjacent copper-cyano chains are joined  through the Cu···Cu interaction forming a three dimensional framework. The site occupancy of cyano ligands C9≡N4 and C8≡N3 is 0.5, each.

### Experimental

A mixture of 3-pyridylacetonitrile (2 mL), cuprous cyanide (0.092 g; 0.1 mmol), strong ammonia water (2 mL) and 8 mL water were sealed in a 23 mL teflon reactor, and the mixture was heated at 443 K for 3 d then cooled to room temperature at a rate of 5 K / h. Yellow crystals were obtained in a yield of 37% based on Cu.

### Refinement

All H atoms were placed in calculated positions and refined using a Riding model, with (C—H= 0.93–0.96 Å), and with *U*_iso_(H) = 1.2 *U*_eq_(C) for methyl H atoms. Two of the cyano ligands are disordered over two sites with occupancies 0.5:0.5.

### Crystal data

**Table:**

[Cu_2_(CN)_2_(C_6_H_7_N)]	*F*(000) = 536
*M**_r_* = 272.27	*D*_x_ = 1.884 Mg m^−^^3^
Monoclinic, *P*2_1_/*c*	Mo *K*α radiation, λ = 0.71073 Å
Hall symbol: -P 2ybc	Cell parameters from 1725 reflections
*a* = 9.3027 (18) Å	θ = 2.3–25.2°
*b* = 12.090 (2) Å	µ = 4.38 mm^−^^1^
*c* = 8.8738 (17) Å	*T* = 296 K
β = 105.927 (2)°	Block, yellow
*V* = 959.7 (3) Å^3^	0.15 × 0.12 × 0.10 mm
*Z* = 4	

### Data collection

**Table:**

Bruker SMART APEX CCD diffractometer	1725 independent reflections
Radiation source: fine-focus sealed tube	1396 reflections with *I* > 2σ(*I*)
graphite	*R*_int_ = 0.035
ω scans	θ_max_ = 25.2°, θ_min_ = 2.3°
Absorption correction: multi-scan (*SADABS*; Bruker, 2004)	*h* = −10→11
*T*_min_ = 0.559, *T*_max_ = 0.668	*k* = −13→14
4802 measured reflections	*l* = −10→10

### Refinement

**Table:**

Refinement on *F*^2^	Primary atom site location: structure-invariant direct methods
Least-squares matrix: full	Secondary atom site location: difference Fourier map
*R*[*F*^2^ > 2σ(*F*^2^)] = 0.042	Hydrogen site location: inferred from neighbouring sites
*wR*(*F*^2^) = 0.118	H-atom parameters constrained
*S* = 1.03	*w* = 1/[σ^2^(*F*_o_^2^) + (0.0603*P*)^2^ + 1.271*P*] where *P* = (*F*_o_^2^ + 2*F*_c_^2^)/3
1725 reflections	(Δ/σ)_max_ = 0.001
107 parameters	Δρ_max_ = 0.76 e Å^−^^3^
2 restraints	Δρ_min_ = −0.77 e Å^−^^3^

### Special details

**Table:**

Geometry. All e.s.d.'s (except the e.s.d. in the dihedral angle between two l.s. planes) are estimated using the full covariance matrix. The cell e.s.d.'s are taken into account individually in the estimation of e.s.d.'s in distances, angles and torsion angles; correlations between e.s.d.'s in cell parameters are only used when they are defined by crystal symmetry. An approximate (isotropic) treatment of cell e.s.d.'s is used for estimating e.s.d.'s involving l.s. planes.
Refinement. Refinement of *F*^2^ against ALL reflections. The weighted *R*-factor *wR* and goodness of fit *S* are based on *F*^2^, conventional *R*-factors *R* are based on *F*, with *F* set to zero for negative *F*^2^. The threshold expression of *F*^2^ > σ(*F*^2^) is used only for calculating *R*-factors(gt) *etc*. and is not relevant to the choice of reflections for refinement. *R*-factors based on *F*^2^ are statistically about twice as large as those based on *F*, and *R*- factors based on ALL data will be even larger.

### Fractional atomic coordinates and isotropic or equivalent isotropic displacement parameters (Å^2^)

**Table:**

	*x*	*y*	*z*	*U*_iso_*/*U*_eq_	Occ. (<1)
C1	0.3796 (8)	0.8237 (6)	−0.1465 (8)	0.085 (2)	
H1A	0.4815	0.8216	−0.0832	0.127*	
H1B	0.3695	0.8783	−0.2273	0.127*	
H1C	0.3520	0.7525	−0.1933	0.127*	
C2	0.2797 (3)	0.8529 (3)	−0.0463 (4)	0.0574 (13)	
C3	0.1740 (4)	0.7751 (2)	−0.0325 (3)	0.0514 (12)	
H3	0.1677	0.7076	−0.0843	0.062*	
N1	0.0778 (3)	0.7981 (2)	0.0587 (3)	0.0464 (9)	
C4	0.0872 (3)	0.8990 (2)	0.1361 (4)	0.0539 (12)	
H4	0.0228	0.9144	0.1971	0.065*	
C5	0.1929 (4)	0.9768 (2)	0.1222 (4)	0.0705 (16)	
H5	0.1992	1.0442	0.1740	0.085*	
C6	0.2891 (4)	0.9537 (3)	0.0310 (5)	0.0703 (16)	
H6	0.3599	1.0057	0.0218	0.084*	
C7	−0.2726 (5)	0.7996 (4)	0.2481 (5)	0.0447 (11)	
C8	−0.0191 (5)	0.5420 (3)	0.0191 (5)	0.0479 (10)	0.50
C9	−0.4653 (6)	0.9731 (5)	0.4697 (6)	0.0674 (14)	0.50
Cu1	−0.07682 (7)	0.68136 (4)	0.07839 (7)	0.0473 (2)	
Cu2	−0.35513 (8)	0.88478 (6)	0.37450 (8)	0.0680 (3)	
N2	−0.2093 (5)	0.7508 (4)	0.1768 (5)	0.0576 (11)	
N3	−0.0191 (5)	0.5420 (3)	0.0191 (5)	0.0479 (10)	0.50
N4	−0.4653 (6)	0.9731 (5)	0.4697 (6)	0.0674 (14)	0.50

### Atomic displacement parameters (Å^2^)

**Table:**

	*U*^11^	*U*^22^	*U*^33^	*U*^12^	*U*^13^	*U*^23^
C1	0.069 (4)	0.102 (5)	0.097 (5)	−0.027 (4)	0.045 (4)	−0.012 (4)
C2	0.052 (3)	0.061 (3)	0.058 (3)	−0.013 (3)	0.012 (3)	−0.001 (3)
C3	0.057 (3)	0.051 (3)	0.050 (3)	−0.010 (2)	0.020 (2)	−0.008 (2)
N1	0.056 (2)	0.040 (2)	0.047 (2)	−0.0042 (18)	0.0218 (18)	−0.0066 (17)
C4	0.066 (3)	0.040 (3)	0.052 (3)	0.010 (2)	0.012 (2)	−0.004 (2)
C5	0.071 (4)	0.043 (3)	0.089 (4)	−0.005 (3)	0.008 (3)	−0.015 (3)
C6	0.066 (4)	0.046 (3)	0.093 (4)	−0.017 (3)	0.011 (3)	0.000 (3)
C7	0.052 (3)	0.038 (3)	0.050 (2)	0.0047 (19)	0.025 (2)	−0.0047 (19)
C8	0.059 (3)	0.038 (2)	0.057 (2)	0.0056 (19)	0.034 (2)	−0.0012 (19)
C9	0.073 (3)	0.069 (3)	0.066 (3)	0.026 (3)	0.029 (3)	−0.009 (3)
Cu1	0.0561 (4)	0.0377 (4)	0.0583 (4)	0.0040 (2)	0.0328 (3)	−0.0070 (2)
Cu2	0.0727 (5)	0.0675 (5)	0.0772 (5)	0.0212 (4)	0.0429 (4)	−0.0155 (4)
N2	0.067 (3)	0.050 (3)	0.068 (3)	0.007 (2)	0.038 (2)	−0.008 (2)
N3	0.059 (3)	0.038 (2)	0.057 (2)	0.0056 (19)	0.034 (2)	−0.0012 (19)
N4	0.073 (3)	0.069 (3)	0.066 (3)	0.026 (3)	0.029 (3)	−0.009 (3)

### Geometric parameters (Å, °)

**Table:**

C1—C2	1.493 (6)	C5—H5	0.9300
C1—H1A	0.9600	C6—H6	0.9300
C1—H1B	0.9600	C7—N2	1.140 (5)
C1—H1C	0.9600	C7—Cu2	1.839 (4)
C2—C3	1.3900	C8—N3^i^	1.158 (8)
C2—C6	1.3900	C8—C8^i^	1.158 (8)
C3—N1	1.3900	C8—Cu1	1.886 (4)
C3—H3	0.9300	C9—N4^ii^	1.150 (9)
N1—C4	1.3900	C9—C9^ii^	1.150 (9)
N1—Cu1	2.057 (2)	C9—Cu2	1.838 (5)
C4—C5	1.3900	Cu1—N2	1.891 (4)
C4—H4	0.9300	Cu1—Cu2^iii^	2.8364 (10)
C5—C6	1.3900	Cu2—Cu1^iv^	2.8364 (10)
			
C2—C1—H1A	109.5	C5—C6—C2	120.0
C2—C1—H1B	109.5	C5—C6—H6	120.0
H1A—C1—H1B	109.5	C2—C6—H6	120.0
C2—C1—H1C	109.5	N2—C7—Cu2	173.8 (5)
H1A—C1—H1C	109.5	N3^i^—C8—C8^i^	0.0 (5)
H1B—C1—H1C	109.5	N3^i^—C8—Cu1	178.1 (5)
C3—C2—C6	120.0	C8^i^—C8—Cu1	178.1 (5)
C3—C2—C1	117.6 (3)	N4^ii^—C9—C9^ii^	0.0 (5)
C6—C2—C1	122.4 (3)	N4^ii^—C9—Cu2	178.9 (8)
C2—C3—N1	120.0	C9^ii^—C9—Cu2	178.9 (8)
C2—C3—H3	120.0	C8—Cu1—N2	142.80 (19)
N1—C3—H3	120.0	C8—Cu1—N1	109.28 (15)
C4—N1—C3	120.0	N2—Cu1—N1	107.10 (16)
C4—N1—Cu1	120.73 (15)	C8—Cu1—Cu2^iii^	81.38 (15)
C3—N1—Cu1	119.27 (15)	N2—Cu1—Cu2^iii^	79.83 (14)
N1—C4—C5	120.0	N1—Cu1—Cu2^iii^	132.57 (9)
N1—C4—H4	120.0	C9—Cu2—C7	169.9 (2)
C5—C4—H4	120.0	C9—Cu2—Cu1^iv^	113.40 (17)
C6—C5—C4	120.0	C7—Cu2—Cu1^iv^	76.72 (15)
C6—C5—H5	120.0	C7—N2—Cu1	170.8 (5)
C4—C5—H5	120.0		

Symmetry codes: (i) −*x*, −*y*+1, −*z*; (ii) −*x*−1, −*y*+2, −*z*+1; (iii) *x*, −*y*+3/2, *z*−1/2; (iv) *x*, −*y*+3/2, *z*+1/2.
